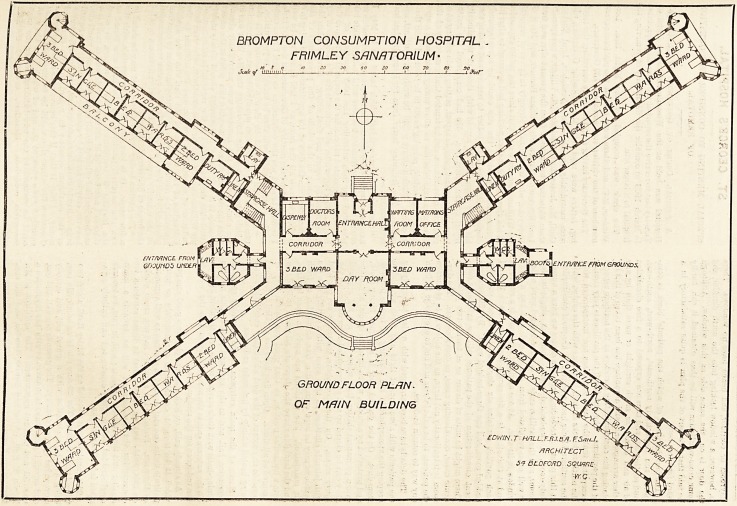# The Sanatorium at Frimley for Brompton Consumption Hospital

**Published:** 1904-06-25

**Authors:** 


### THE SANATORIUM AT FRIMLEY FOR BROMPTON CONSUMPTION HOSPITAL.

THIS institution, which has been erected at a cost of £70,000 for the purpose of treating such cases of phthisis as afford hope of permanent benefit from residence in a sanatorium, contains accommodation for eighty-eight patients. The block in which the patients are placed is shaped like a St. Andrew's Cross, or rather the four pavilions of which the block consists are arranged in this form, and at the point where the ends of the pavilions would meet the cross has been extended so as to find room for entrance hall to the north and a day-room with a fine circular bay to the south. At one side of the former are the waiting-room and matron's office, and on the other side are the doctor's office and dispensary. East and west of the day-room and facing south are two three-bedded wards. Springing from the central portion of the block east and west are the sanitary annexes, which contain bathrooms, closets, lavatories, boot-rooms, and entrance porch for use of patients going to or from the grounds. These annexes are very well arranged, and are efficiently cut off from the main building, and yet they occupy central and convenient positions for the patients.

The three-bedded rooms have two windows facing south, but their east and west walls are merely party walls of dayroom or corridor, and hence have no windows. There is, however, a window looking into the north corridor, and the door is on the same side as this window. Although some cross-ventilation will be thus obtained it can hardly be said that this part of the plan is quite satisfactory in a ward for three beds.

The four pavilions of which the patients' block is composed run respectively north-east, north-west, south-east, and south-west, and thus are exposed to a free circulation of air and obtain as much sunlight or more than by any other arrangement; and advantage has been taken of this by placing the communicating corridors at the northern aspects of the pavilions. The division of the pavilions is very similar excepting that the main staircases are incorporated with the northern blocks at their junctions with the centre, while the southern blocks have a section of fire-proof construction at this point. Proceeding from the centre we have a nurses' duty-room, a two-bedded room, then six single-bedded rooms, and the pavilion ends in a three-bedded ward which is well cross-ventilated from end to end, and has a large window in its side. At one angle of this ward is a sun-room and at the other is a fire-escape staircase. Each floor of each pavilion contains six single-bedded rooms, one two-bedded room, and one three-bedded ward. By this arrangement classification of the patients will be extremely easy. There might be units of eleven or of twenty-two patients—acute cases, convalescing cases, or convalescents. The dayroom is intentionally not large, as the object is to encourage the patients to be out-of-doors and not to encourage them in any way to remain in-doors.

The details have been arranged with a view to supplying the patients with abundance of light and fresh air, and to keeping the atmosphere in the building as free as possible from dust. On the ground floor is a terrace facing south, and protected by awnings, which have the special advantage over a permanent shelter, that when they are not required they can be closed up and so prevented from darkening the lower rooms. On the floor above, the rooms are provided with jalousie shutters, so that even with a driving rain the windows may be kept open and a free circulation of fresh air maintained. The windows are made to open inwards, and the fanlights above the doors are all made to let down into a horizontal position, so that good ventilation may be insured at all times. Open fireplaces are avoided as much as possible on account of the dust which they entail, the rooms being heated mainly by hot-water radiators.

North of the main entrance is the assembly room or recreation hall, and to the east and west of this are the dining-halls with their serving-rooms near the main kitchen. To the extreme east is the nurses' home, and to the west is the residence for the medical staff.

As already said, we look upon the plan as a good one provided it be thought desirable to accommodate so many patients under one roof, even if in sections so well separated as these are. Probably the architect had not much choice on this point, but we believe that in these sanatoria for consumption the development will be towards a much smaller unit than eleven patients; will, in fact, be towards single-bedded huts properly constructed and entirely separated from their fellows.

This sanatorium is situated on the Bisley Road, about two miles from Frimley. The 20-acre site is an admirable one, being at a high elevation, with a gravel soil, and entirely surrounded by open country. The grounds are well laid-out, and especial pains have been taken to give the institution a cheerful and pleasant appearance. The architect is Mr. Edwin T. Hall, of Bedford Square, London, and the contractors are Messrs. Holliday and Greenwood, of Brixton.

## Figures and Tables

**Figure f1:**